# Men’s involvement in women’s abortion-related care: a scoping review of evidence from low- and middle-income countries

**DOI:** 10.1080/26410397.2022.2040774

**Published:** 2022-03-24

**Authors:** Joe Strong

**Affiliations:** PhD Researcher, Department of Social Policy, London School of Economics and Political Science, London, UK. *Correspondence:* j.strong3@lse.ac.uk

**Keywords:** abortion, men, masculinities, LMICs, reproduction, sexual and reproductive health and rights

## Abstract

Men’s involvement in abortion is significant, intersecting across the individual, community and macro factors that shape abortion-related care pathways. This scoping review maps the evidence from low- and middle-income countries relating to male involvement in abortion trajectories. Five databases were searched, using search terms, to yield 7493 items published in English between 01.01.2010 and 20.12.2019. 37 items met the inclusion criteria for items relating to male involvement in women’s abortion trajectories and were synthesised using an abortion-related care-seeking framework. The majority of studies were conducted in sub-Saharan Africa and were qualitative. Evidence indicated that male involvement was significant, shaping the ability for a woman or girl to disclose her pregnancy or abortion decision. Men as partners were particularly influential, controlling resources necessary for abortion access and providing or withdrawing support for abortions. Denial or rejection of paternity was a critical juncture in many women’s abortion trajectories. Men’s involvement in abortion trajectories can be both direct and indirect. Contextual realities can make involving men in abortions a necessity, rather than a choice. The impact of male (lack of) involvement undermines the autonomy of a woman or girl to seek an abortion and shapes the conditions under which abortion-seekers are able to access care. This scoping review demonstrates the need for better understanding of the mechanisms, causes and intensions behind male involvement, centring the abortion seeker within this.

## Résumé

La participation des hommes à l'interruption de grossesse est importante, et touche à des facteurs individuels, communautaires et généraux qui modèlent les parcours de soins relatifs à l'avortement. Cet examen de portée recense des données provenant de pays à revenu faible ou intermédiaire en rapport avec la participation des hommes aux parcours d'avortement. Cinq bases de données ont fait l'objet de recherches, à l'aide de termes de recherche, pour obtenir 7493 articles publiés en anglais entre le 01.01.2010 et le 20.12.2019. Trente-sept articles réunissaient les critères relatifs à la participation masculine dans les parcours de l'avortement des femmes et ont été synthétisés à l'aide d'un cadre de recherche de soins relatifs à l'avortement. La majorité des études avaient été menées en Afrique subsaharienne et étaient qualitatives. Les données indiquaient que la participation masculine était importante, et qu'elle influençait la capacité d'une femme ou d'une jeune fille à révéler sa grossesse ou sa décision d'avorter. Les hommes en tant que partenaires étaient particulièrement influents, contrôlant les ressources nécessaires pour l'accès à l'avortement, et prodiguant ou retirant leur soutien à l'avortement. Le déni ou le rejet de la paternité était une étape critique dans le parcours de beaucoup de femmes pour avorter. La participation des hommes peut être à la fois directe et indirecte. Les réalités contextuelles peuvent faire de la participation masculine une nécessité plutôt qu'un choix. L'impact de la participation des hommes (ou de leur manque de participation) sape l'autonomie dont dispose une femme ou une jeune fille pour demander un avortement et façonne les conditions dans lesquelles les femmes souhaitant interrompre leur grossesse peuvent avoir accès aux soins. Cette étude de portée démontre la nécessité de mieux comprendre les mécanismes, les causes et les intentions derrière la participation des hommes, en se centrant sur la personne qui demande l'avortement.

## Resumen

La participación de los hombres en la trayectoria de aborto es significativa; se entrecruza con factores individuales, comunitarios y macro que influyen en los trayectos relacionados con los servicios de aborto. Esta revisión de alcance mapea la evidencia de países de bajos y medianos ingresos relacionada con la participación de los hombres en las trayectorias de aborto. Se utilizaron términos de búsqueda para realizar una búsqueda en cinco bases de datos, que produjo 7493 ítems publicados en inglés entre 01.01.2010-20.12.2019; 37 ítems reunieron los criterios de inclusión para ítems relacionados con la participación de hombres en las trayectorias de aborto de las mujeres y fueron sintetizados utilizando un marco de búsqueda de servicios relacionados con el aborto. La mayoría de los estudios fueron realizados en África subsahariana y fueron cualitativos. La evidencia indicó que la participación de los hombres era significativa, ya que influía en la capacidad de la mujer o niña para divulgar su decisión sobre su embarazo o aborto. Los hombres como parejas eran particularmente influyentes, ya que controlaban los recursos necesarios para el acceso a los servicios de aborto y proporcionaban o negaban el apoyo para un aborto. La negación o el rechazo de la paternidad fue un momento crítico en la trayectoria de aborto de muchas mujeres. La participación de los hombres en la trayectoria de aborto puede ser directa e indirecta. Las realidades contextuales pueden tornar la participación de los hombres en el aborto una necesidad, y no una elección. El impacto de la participación (o falta de participación) de los hombres socava la autonomía de una mujer o niña para buscar un aborto e influye en las condiciones bajo las cuales las personas que buscan un aborto son capaces de acceder a los servicios de aborto. Esta revisión de alcance demuestra la necesidad de entender mejor los mecanismos, causas e intenciones detrás de la participación de los hombres, centrando a la persona que busca el aborto en este marco.

## Introduction

Pathways to abortion-related care can be complex and iterative and are affected across individual, community, national and international contexts. Men have a significant impact on the sexual and reproductive health and rights (SRHR) of others. In 1994, the International Conference on Population and Development recognised this by outlining the need for further engagement with men and boys in its *Programme for Action.*^1^ It aimed to grapple with how men contribute to shaping the contextual conditions under which women and girls have to navigate their SRHR.^2^ Following the *Programme for Action*, there was an increase in policy and programming aimed at engaging men, particularly as “partners” in SRHR.^3^ These have been particularly focused in low- and middle-income country (LMIC) settings.^4–6^

Autonomous and free access to safe abortions remains a major concern across the world, particularly where resources and capabilities to provide safe abortion services are limited.^7^^,^^6^ Of less and least safe abortions, 97% are estimated to occur in LMIC contexts,^7^ and contribute to higher rates of complications than safer abortions.^6^ The conditions under which these abortions occur are shaped by intersecting abortion-specific, individual, and sub-/national factors,^8^ including structurally violent, gendered power systems,^9^ that implicate men in a person’s abortion-related care trajectory.

Engaging men is a critical mechanism to challenge and reshape the normative environment that shapes abortion.^10–12^ However, it risks increasing men’s power and control by inserting them as actors into abortion trajectories.^13–15^ Studies among abortion-seekers have consistently referenced the role and influence of men at the structural level and the individual level.^16^ Evidence from the multi-country International Men and Gender Equality Survey (IMAGES)^17^ emphasised that men were “substantially” involved in abortion decisions if a pregnancy was disclosed,^17^ while evidence from abortion-seekers illustrates that a large proportion of women and pregnant people cite that their (male) partner was a reason for their decision to seek care.^18^ This includes the potential benefits of partner involvement within care decisions, such as emotional, material, and financial support.^8–19^

Previous evidence syntheses highlight that abortion care is linked to broader economic, social, and political structures,^20–22^ focused on abortion and post-abortion care,^23^^,^^24^ as well as specifically self-management.^25^^,^^26^ Altshuler et al.’s^27^ systematic review on the roles of men in abortion-related care was primarily focused on “male partners”, with studies ranging across 1985–2012. Studies were excluded if abortions were done outside of legal frameworks, due to fetal indications, or where men’s involvement was considered coercive. They found that male partners were involved in four areas: presence at medical facilities, participation in pre-abortion counselling, presence in the procedure room or while a partner obtained a medical abortion, and participation in post-abortion care.

The review emphasises the role of men as significant. However, considering the increasing need to engage men beyond their role as partners,^16^^,^^28^ in order to fully grapple with the normative environments and conditions under which women and pregnant people obtain care,^1^^,^^29^^,^^30^ a broader scoping review of men’s involvement in abortions is both relevant and necessary.

## Methods

This scoping review aims to map the recent evidence of men’s involvement in abortion-related care trajectories. It understands involvement to be both direct – where men are present in the decision-making process – and indirect – where men exert influence and shape an abortion trajectory without being actively involved in the decision-making process. This includes understanding how men have been included in research samples, methods used, and geographic foci, in order to consider how future research can develop the evidence. A scoping review is the most appropriate method, as it produces an overview of evidence rather than clinical or policy guidelines, which require a systematic review.^31^ The protocol for this study is available.^32^

This review utilises the abortion trajectories framework, developed by Coast et al.,^8^ in order to situate men’s involvement. The framework establishes three intersecting domains that shape the trajectory of abortion, from the decision to abort, the ability to access care, choice of method, and outcomes of care. The first – abortion-specific experiences – begins with pregnancy awareness and includes time-orientated factors that shape the experience of care. The second – individual context – considers the characteristics and relations (e.g. interpersonal network) that influence whether a woman obtains abortion-related care. The final domain – (inter)national and sub-national contexts – includes the norms and contextual conditions within which an individual and their abortion are situated.

### A note on terminology

Findings in this study refer to men and women. This reflects the language that was used within the included studies. It is not used to exclude the reality that people of any gender can and do become pregnant and require abortion care.^33^^,^^34^

### Inclusion and exclusion

Articles were included if they met all the inclusion criteria: published between 01.01.2010 and 20.12.2019, research on humans, English language, peer-reviewed, focused on abortion, include men as the sample or evidence on men, or evidence on male providers.

The shifting landscape of abortion-related care trajectories, impacted by new technologies, methods, and legal changes, made a short publication date range suitable.^35^^,^^36^ Moreover, the only systematic review of men and abortion included publications between 1985 and 2012.^27^ This evidence mapping aims to build on the current evidence on men’s involvement, whilst ensuring the studies included are relevant to the current abortion landscape.

In preference of depth over breadth of evidence, non-article publications (e.g. published abstracts) were excluded. Studies were included irrespective of geo-political categorisation, labelling them as either a high-income country (HIC) or LMIC study *post hoc*, using World Bank classifications.*

### Databases and search strategy

Five social science databases (EMBASE, PsychINFO, MEDLINE (Ovid), CAB Direct, CINAHL) were searched using a web of connecting terms, including MeSH terms for MEDLINE (Ovid) and EMBASE where applicable (Table 1). These search terms were designed to reflect the focus on male involvement in women’s and girls’ abortion trajectories. The dates, language and peer-review were constrained in all journal searches. For EMBASE, PsychINFO and MEDLINE (Ovid), constraints to ensure only studies involving humans were used.

**Table 1.** Outline of search terms for EMBASE, PsychINFO, MEDLINE (Ovid), CAB Direct, CINAHL.

Abortion/pregnancy search terms	Gender/men search terms	Pathways and trajectories search terms	Involvement search terms
Abortion* Termination* (Menstru* and regulat*) Antenatal	Man Men Male Masculin* Adolescen* Boy*	Pathw* Passage* Rout* Course* Traject* Direction*	Influen* Involv* Support* Participat*

Note: The * indicates truncated search terms.

((Abortion* or termination* or (menstru* and regulat*) or antenatal) and (man or men or male or masculin* or adolescen* or boy or boys) and (pathw* or passage* or rout* or course* or traject* or direction* or influen* or involv* or support* or participat*)).

**Figure 1.**
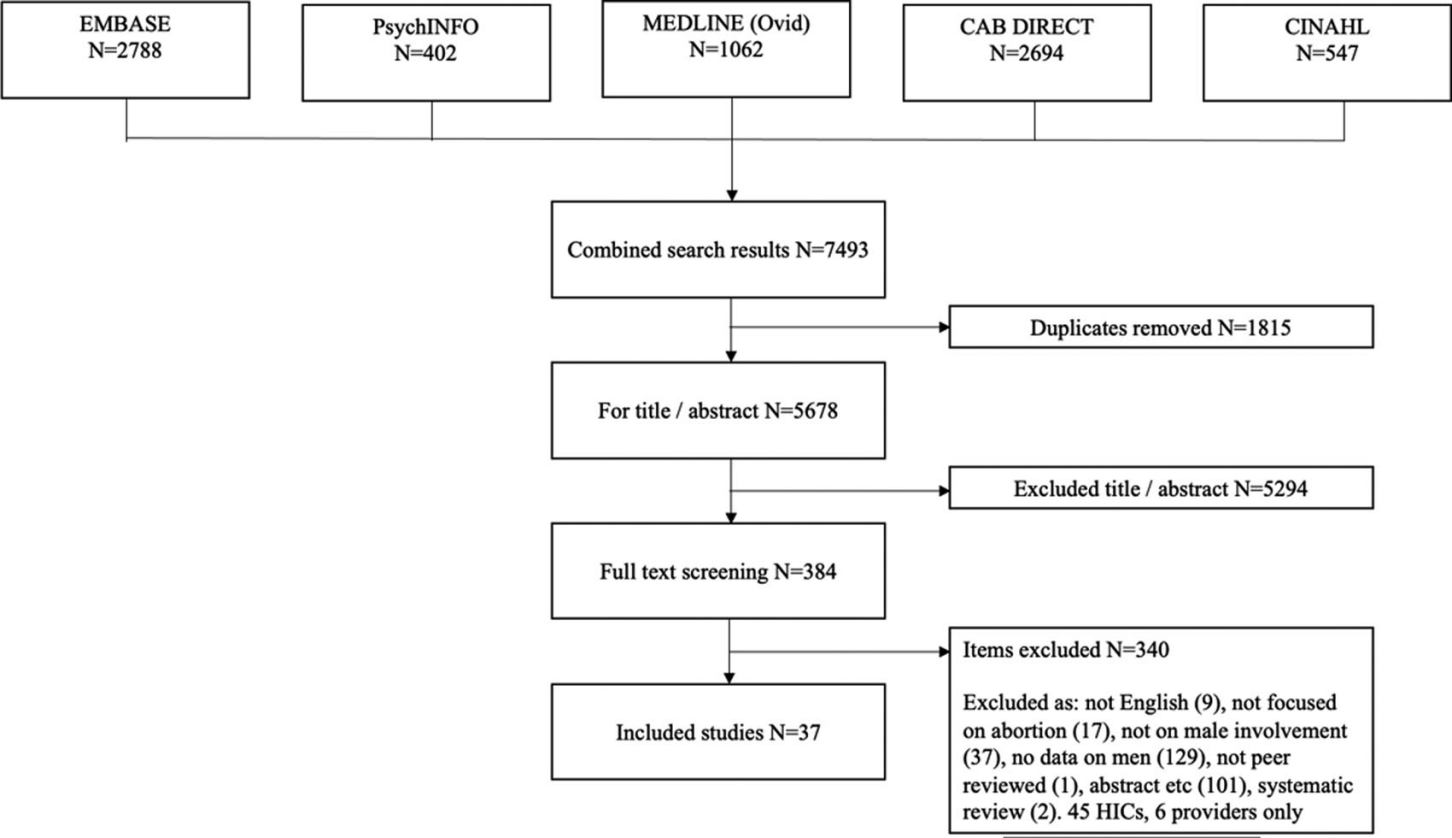
Flow diagram of screening process.

The author removed all duplicates before screening the titles and abstracts (TIAB) of articles, excluding any that did not indicate meeting the full set of inclusion criteria. A full-text screening of all included articles was then conducted. After a combined result of 7493 articles, 1815 were excluded as duplicates, 5678 were screened on TIAB (see Figure 1). A 5% sub sample of studies included for TIAB was cross checked by a research assistant, Clara Opoku Agyemang (see Methodological limitations).

Studies with a focus on abortion were included if they had men in the sample, or if reference was made to men’s involvement, regardless of whether the sample included men. The decision was made to be more inclusive for full-text screening, to reflect that the gender of parents, partners, friends, and family members might not be specified in the abstract. 384 articles were taken from TIAB to full-text screening. Of these, 9 were not in English, 17 not focused on abortion, 37 did not include male involvement, 129 had no evidence on men, 1 was not peer-reviewed, 101 were abstracts, posters, etc., and 2 were systematic reviews.

A total of 88 studies were included across geographies. The decision to separate the scoping review between LMICs (*n* = 43) and high-income countries (*n* = 45) reflects the nature of policy and research within these geo-political domains. LMICs are more directly impacted by global health discourse and rhetoric, illustrated by the increasing focus on men in global health policies within these contexts – for example in the recent maternal mortality reduction initiatives in LMICs.^37^^,^^38^ Thus, this evidence mapping can engage with specific audiences and political currents that shape the research agenda in LMICs.

Of the 43 studies taken to full-text screening, six of these related solely to abortion providers, which was an initial component of interest. However, the data extraction process indicated that the research on providers was less developed with regard to involvement. This scoping review, therefore, presents the results of men’s involvement exclusive of men who work within medicalised spaces. Thirty-seven studies have been included for this review.

Data extraction was conducted on Endnote X9.^39^ This followed a codebook that had categories for background information (author, date, study setting, country(ies) of study), study information (methods, sample, recruitment sites), and primary outcomes of interest based on the abortion trajectories framework (abortion-specific experiences, individual context, (inter)national and sub-national contexts). These data were extracted solely by the author (JS). A copy of the extraction codebook is available on request from the author and the abridged summary table can be found in Appendix. A quality assessment of the included studies was not conducted, as this is not standard protocol for a scoping review.^31^ The quality of the studies is not essential when mapping the existing evidence and gaps.

### Methodological limitations

The inclusion criteria reflect the constraints of the research team, and therefore, only studies in English were included. This scoping review is, therefore, limited to English-language studies and does not reflect the full scope of evidence on men’s involvement in abortion published in other languages or on platforms outside of the databases used.

Due to resource constraints, the majority (95%) of studies in the evidence mapping were screened by the author, with a randomly selected 5% sub-sample blind double-screened by a research assistant (RA). “Blind” refers to neither reviewer knowing the results of the other’s screening until after both are completed. The purpose of this was to identify any systematic subjective biases in the screening process by the author, through emergent discrepancies in results. Within the 5% sub-sample, if minor (<1%) discrepancies were found, these would be discussed and an outcome for each agreed upon. Efforts were made prior to conducting the screening to ensure that both the researcher and RA felt comfortable with the review and the process of screening, so as to facilitate a discussion space for any discrepancies. If there were major (>1%) discrepancies, or systematic differences in which the same type of discrepancy over an exclusion/inclusion criterion emerged, then a larger sample of the studies would be drawn for a second blind review. No discrepancies did emerge during the sub-sample screening.

This sub-sample blind screening process does not negate the possible bias as a result of a single author screening but does aid in mitigating these biases. Moreover, it remains possible that papers were missed due to language constraints and during the screening. A previous systematic review on men and abortion similarly identified that the lack of detail in abstracts and gender disaggregation might have led to studies being erroneously excluded.^27^ The decision to take more texts to full screening was aimed to mitigate that, as well as the need for a scoping review, as opposed to a systematic review, to allow for more flexibility in the methodological limitations.^31^ Data extraction was conducted by one individual, due to constraints. It is possible that during data extraction, some data and evidence were missed, however, re-reviewing each full text for a second extraction review aimed to mitigate this possibility.

## Results

The majority of studies (26/37) were qualitative, with the remainder quantitative (5/37) or mixed-method/unclear (6/37). Study contexts were predominantly located in sub-Saharan Africa and South Asia. The largest sample size of the studies used Demographic and Health Survey data, which surveyed 3848 women in Kyrgyzstan^40^ and was the only nationally representative sample used. 23/37 studies used samples recruited in or referred from health facility lists (including pharmacies, abortion providers, post-abortion care facilities). The remainder were recruited through community networks/household surveys (10/37) or from schools or universities (4/37). See Appendix for included studies.

The results are divided into the three domains identified in the trajectories of abortion-related care framework: abortion-specific experiences, individual contexts, and (inter)national/sub-national contexts.^8^ This allows for the scoping of men’s involvement to be mapped onto abortion trajectories.

### Abortion-specific experiences

The majority of studies (27/37) reported on abortion-specific experiences, ranging from men’s direct and indirect involvement in decisions and responses to pregnancy disclosure, support for/against abortion, (non-)provision of material and physical resources, and access to abortion providers or methods.

Disclosure is a critical component of an abortion-related care trajectory, as it can impact whether and how a woman is able to obtain an abortion.^8^ With the exception of a study of young men in the Philippines,^41^ all evidence on the experience of disclosure was from women who had sought abortions, or studies where men were a secondary sample of interest. Women who had either sought abortion care or post-abortion care at a facility in Lusaka, Zambia, reported that the fear of disclosure also included fear of partner interference in the pregnancy or abortion decision, and fear of repercussions from fathers.^42^

The fear of potential responses to disclosure also shaped the conditions under which women and girls made pregnancy and abortion decisions. A study with women aged 15–49 in Ghana highlighted how fears of being disowned, abused, or ejected by parents (not disaggregated between mothers and fathers) impacted their pregnancy disclosure and subsequent abortion decision-making.^43^ Women in a study of abortion care-seekers in Ghana reported that fear of disclosure, including to partners, influenced their decision to self-manage,^44^ and women in a second study interviewing men and women in Ghana reported similar fears of disclosure.^45^ Among women in Brazil, fear of disclosing induced abortions related to their partner’s potential reaction, whereas disclosure of a miscarriage led to fear of family reactions.^46^ In one study, men and boys also reported fears of disclosure of a pregnancy impacting their decisions and involvement in an abortion. Respondents in a qualitative study of attitudes towards abortion in the Philippines reported that their interference and pressuring for their partner to an obtain an abortion stemmed from their fears to disclose their partner’s pregnancy.^41^

The most common evidence of men’s involvement in abortion-specific experiences was in the provision of material and physical resources. Financial provision was important in shaping the type of abortion that women obtained, as well as impacting women’s choice whether to disclose their pregnancies. In a study in Zambia, 50.4% of respondents reported that they had to involve men in their decisions in order to obtain the necessary finances to cover the costs of care.^47^ A qualitative study of 112 women who had obtained abortions or post-abortion care in Zambia found that women’s disclosure was determined by their desires to maintain autonomy over their decision-making; for those that involved men in their pregnancy disclosure, this included men paying for the cost of care.^42^ Adolescents in a study of reproductive decisions in Mexico City reported that their partner’s support for their abortions was conditional, and that the latter’s provision of resources impacted women’s and girls’ choice of abortion care.^48^

The provision of resources was also interlinked with the provision of support for/against an abortion decision. In a qualitative study of 80 women in Nairobi, men were reported as exerting pressure on the decision-making process, including giving women money to influence them to obtain an abortion, as well as some men pushing for the pregnancy to continue.^49^ A mixed-methods study with 401 women who had obtained abortions in Ghana reported that men utilised their position as “breadwinners” – providers and controllers of financial resources in the household – to pressure women to obtain abortions.^50^

A study of men and women living in the same household in Uganda indicated that men considered their support of abortion to primarily involve the provision of finances for medicine, transportation, food, and costs of potential post-abortion care.^51^ Evidence from men and women in Nigeria similarly found that men (as partners) provided financial, as well as emotional and material, support for women’s abortion-related care, though women also reported that men would give them money as a way of expressing their own desires for a woman to obtain an abortion.^52^

Partners were not always the main sources of finances and resources, nor supportive, and adolescent men in a study in Peru reported that their financial dependence on parents reduced their role in pregnancy decision-making, which was also reported by adolescent women in the study.^53^ An exploration of community perceptions of abortion in Kenya reported that women relied on boyfriends, as well as friends, relatives, and mothers, for financial support.^54^ Moreover, in a qualitative study of 34 unmarried young women seeking abortions in India, only two reported that their partners provided financial support, with the majority citing mothers as supporting their abortion trajectories.^55^

Non-financial support included emotional support, accompaniment, and supporting women’s autonomous decisions. Two studies of abortion experiences among 549 women in India reported that 92% of respondents were supported by their partners, of which 86% reported emotional support and 51% financial support.^56^^,^^57^ In Thailand, women who had experienced complications from abortions reported that finances were an important component of their partners’ support, alongside emotional support, particularly accompanying and telephoning them.^58^ A study of women in Malaysia similarly found that men provided financial support, but also accompanied women and provided moral support, including googling whether abortions were considered a sin under the Islamic faith.^59^

In a study with 1271 unmarried women aged 15–24 in China, 73–85% (variation due to multiple study sites) reported that their partner supported their abortion decisions, particularly by helping them seek care.^60^ Another study of 29 women who had obtained an abortion in China found that men were able to accompany women and were involved in post-abortion family planning decisions.^61^ An evaluation of an intervention to improve knowledge of medical abortion in Cambodia found that men learnt about abortions through newspapers and radio, with four of six men interviewed accompanying their partners for medical abortion and three accompanying for post-abortion care.^62^

Among students in six public secondary schools in Nigeria, 26.8% of the 11% of men who knew a partner was pregnant provided assistance.^63^ In a study with men in northern Ghana, the two main reasons given to support an abortion were for a person to finish schooling or for birth spacing; fewer men supported abortions for unplanned pregnancies.^64^ Men in this study reported buying pharmaceutical and non-pharmaceutical abortion methods to support a partner’s abortion, in order to keep the abortion secret from the community.

Boyfriends were among the people that women in Ghana reported obtained abortion medication for them,^44^ similarly found in a separate study among adolescents in Ghana.^65^ In both studies, women reported being concerned over the safety and efficacy of the medicines. Evidence from women and adolescents who sought abortions or post-abortion care in Zambia included one adolescent reporting that her boyfriend’s brother gave her correct abortion information and provided support through his medical insurance scheme. In a study of medical abortion users and their partners in India, men reported accessing the medical abortion kits on behalf of their partners.^66^ However, the study also reported that key health information on medical abortion was sometimes not passed on from the partner who obtained the kit to the person obtaining an abortion.

Support from men was reported as conditional on their own desired outcomes, and studies also reported that men could be coercive in attaining these. While young women in Mexico, aged 13–17, reported in focus groups that men offered emotional support for their pregnancy decisions, they discussed that these were often in accordance with men’s desired outcomes and not their own.^47^ A study of women in Kenya included a respondent reporting that her husband found a provider to help him induce her abortion without her consent.^67^ In the study, it was reported that almost all women expressed that they disagreed with their partner and feared possible consequences of their pregnancy disclosure (violence, divorce), which led them to seek care without telling their partner. However, some women disclosed their pregnancies in order to obtain financial support for care.

### Individual context

Seventeen studies included evidence relating to the individual context within which a person seeks an abortion. These focused on the partner, family, and community context shaping the perceptions of pregnancy and abortion, denial/rejection of pregnancies.

Denial/rejection of pregnancies was one of the foremost ways that studies reported the context shaped a woman’s abortion trajectory. Rates of pregnancy denial could be high, with a study of 1047 secondary school students in Nigeria reporting that 48.2% of men whose partners were pregnant had denied paternity.^63^ A study of women who had obtained abortions in Ghana reported that being unmarried and in a partnership was a factor in obtaining abortion care, as women reported that they feared their partner could and would abandon them, resulting in their navigation of the stigma of being an unmarried mother.^45^ In a qualitative study of men and women at local universities in Nigeria, women reported that concerns over their partner denying a pregnancy and leaving them without a “responsible” partner influenced decisions to abort.^52^

The impact of partner rejection of a pregnancy was emphasised in a study with women seeking abortions or post-abortion care in Zambia, who reported that their abortion was specifically due to partner rejection, which was also more likely among younger respondents than older.^42^ Moreover, where women reported that their partner was present and knew of their abortion, the majority obtained safe abortions, while those whose partners were absent were predominantly seeking post-abortion care. A mixed-methods study of 15 pregnant adolescents aged 15–19 in Tanzania indicated that the decision to keep a pregnancy was done despite male partner rejection and led to feelings of regret towards becoming pregnant.^68^ Of the 34 adolescents interviewed who had induced abortions in Lusaka, Zambia, 16 reported that their partners rejected or denied paternity and requested them to obtain an abortion.^69^ This rejection of pregnancy included withholding financial support for the pregnancy or future childcare.

The broader individual context also included the attitudes and desires of partners, as well as the living conditions and the relationships of women and girls to their partners and families. Women and girls in Nairobi, Kenya, reported that their partner’s fertility desires meant that some respondents felt pressured into obtaining an abortion.^49^ An analysis of the Kyrgyzstan Demographic and Health Survey, which had a sample of 3848 women aged 15–49, suggests that men’s attitude towards abortion was significantly associated with the likelihood of a woman obtaining an induced abortion.^40^ However, among 142 university students in Ghana, women reported that their own beliefs, including religious beliefs, were important in their abortion decision-making, and that their partner’s and peers’ views were less influential.^70^

A study with 401 women who had obtained abortions in Ghana found that knowledge of the law, occupational status, number of children living, and level of formal education all increased the odds that a women sought consent of male partners in comparison with those that sought consent from “others”, including friends, siblings, and aunts.^50^ Living with parents, particularly fathers, was associated with increased pressure to allow their involvement in abortion decisions among adolescents who had been pregnant at least once in a study in Accra.^71^

Many of the studies that reported on the family context, however, did not disaggregate between type of parent or carer. While studies in Peru,^53^ Ghana,^43^ and Zambia^72^ indicated that the relationship an adolescent or young person had with their parents and family influenced their abortion trajectory, it was not clear if male family members had differing involvement to female family members. In Peru, some male respondents argued that the decisions on pregnancy and abortion were theirs, whilst others supported women’s decisions.^53^ Women and girls described that being younger or less informed was linked to partners taking control of decisions, in addition to describing being coerced to have an abortion by partners and parents.

Evidence also suggests that the type of relationship between partners influenced abortion trajectories, particularly women’s perceptions of their partner as stable and (maritally) committed. In two studies – one in Mexico and one in Sri Lanka – the stability and perceived future of a relationship impacted the abortion trajectory. For women in Mexico, all women whose partners were not involved obtained an abortion.^48^ Among Sri Lankan women, partners who refused to marry or denied paternity also had an impact on the decision to obtain an abortion.^73^ In addition, respondents cited the involvement of their brothers in pressuring them to obtain an abortion, if they were pregnant while unmarried. A study of pregnancy reactions among adolescents who recently had an abortion in Ghana suggested that a partner being a student or unemployed could lead to them suggesting an abortion, and respondents also cited men’s ability to deny a pregnancy as significant.^65^

### (Inter)national and sub-national contexts

Seven studies reported on how the (inter)national and sub-national contexts are both shaped and maintained by men, as well as having an influence on men’s involvement in abortion decisions. These studies primarily focused on the role of men in operationalising social norms around abortion in their response to a pregnancy or abortion. Community leaders in a study in the Democratic Republic of the Congo, who were all male, reported that women who sought abortions would be actively stigmatised, isolated, and/or forced to leave their communities.^74^ However, in instances where a women’s partner was abusive, alcoholic, or unemployed, or where there were financial difficulties, community leaders were more supportive of abortions, as well as considering themselves responsible for post-abortion care. Men could utilise cultural norms to involve themselves in abortion decision-making. A study of the national discourses around masculinities and abortion in South Africa revealed that the “New Man” discourse – referring to men who considered themselves committed, caring, and loving to their partners and family – was a mechanism through which men reported being supportive of pregnancies to order to dissuade partners from obtaining an abortion.^75^

Attitudes towards abortions that drew on, and bolstered, prevailing social and cultural norms were complex and varied. In a study of abortion in Uganda, men responded that they were generally not supportive of women having an abortion, aligning their beliefs with prevailing socio-cultural norms, which shaped their decisions to provide support or finances in the event of a pregnancy.^51^ Young Filipino men discussed in focus groups how they viewed abortion as a “sin” and that, in accordance with their normative environment, they were not supportive of women obtaining care.^41^ However, in-depth interviews indicated that these men considered abortions acceptable under certain conditions. Among men in Ghana, abortion was similarly labelled as a “sin” and unacceptable by community norms, although these norms were also operationalised by men in focus groups to discuss how stigmatised pregnancies were a reason to encourage an abortion.^64^

While men in a study in Kenya were reported to consider women who had abortions as not “wife material”, a norm which forced some women to relocate in order to obtain care, men and women in the study also reported that abortions were increasingly normalised in the community.^54^ Community norms could also be enacted to minimise men’s involvement. In a study of parental attitudes towards induced abortion in Nigeria, mothers reported that it was a social necessity that decisions be between mother and daughter, while fathers suggested that their role was as breadwinners.^76^

## Discussion and conclusion

Studies highlight the potentially significant – and diverse – role that men and boys can have in women and pregnant people’s abortion trajectories across low- and middle-income settings. The evidence emphasises that men’s involvement was present across abortion-specific experiences, the individual context of an abortion seeker, and the community context. This review complements broader evidence on the role of men in sexual and reproductive health, which has highlighted their ability to influence fertility and contraceptive decisions,^77–82^ and shape care-seeking through financial gatekeeping,^83^ in addition to providing positive support for partners.^14^ Similarly to this broader evidence on men and SRHR,^84–86^ this review highlights the diverse implications of men’s involvement in abortion trajectories.

Partners – boyfriends, husbands, sexual partners, etc. – are the men who are most often included in study samples or referred to by women and pregnant people, mirroring the focus on partners in global health discourse.^87^ However, included studies also indicated that other male relations – including fathers and brothers – can be important, as well as how men – such as community leaders – are able to shape the normative structures that can govern abortion trajectories. While parents were referenced in numerous studies, this was not always disaggregated to investigate whether there were differences between parental roles of fathers and mothers, as well as other guardians or carers.

In these studies, evidence on men’s involvement in abortion trajectories was particularly prevalent for abortion-specific experiences and highlighted how this intersected across experiences of disclosure and financial and emotional support. The real or perceived expectations of how a partner, or sometimes father, would react to a pregnancy had an impact on a woman’s decision over pregnancy or abortion disclosure. The most frequently reported area of men’s involvement in abortion-specific experiences in studies, however, came in the control and provision of resources. This was referred to in studies across different contexts, emphasising the widespread nature of men’s control of resources and finances. Women are, therefore, made to navigate the complexities of disclosing their abortion intention to possible negative reactions, or having the resources and finances necessary for transport or facility costs limited. The included studies suggest that men are integral to creating the conditions that shape the ability of women and pregnant people to make free and autonomous choices on their abortion intention and desired care pathway.

Studies also provide evidence of how men shape both the individual contexts and the broader environments within which women and pregnant people seek care. The relationship between a woman or pregnant person and their partner, as well as the age of an adolescent, impacted their decision-making and the trajectory of their abortion. Studies with a sample of men most frequently provided evidence of men’s roles in shaping the broader discourse of abortion, upholding and (re)producing contextual norms. These norms create the conditions under which pregnancies can be stigmatised, resulting in women or pregnant people seeking abortions, or that require abortions to be conducted privately away from institutions or public facilities. These norms and contexts were linked to the denial or rejection of paternity, which in turn (re)shapes the contextual conditions that impact an abortion trajectory.

It is not possible to ascertain the extent to which this indirect involvement from men, particularly their involvement in shaping the broader conditions of care, shapes the explicit choices and experiences of women and pregnant people seeking abortions. Few studies can make clear in the evidence whether men’s involvement was sought by women or pregnant people as part of their free and autonomous choice for care, or out of necessity for information, resources, and to make the context of their abortion more acceptable. Moreover, biases of the sampling frames are not (always) clear in the current studies. For example, men who are sampled often accompanied their partners and might be more supportive by virtue of this, and abortion experiences outside of facilities where participant recruitment occurs are less represented.

In addition to the methodological considerations, this scoping review is limited in its capacity to synthesise evidence to make policy- and clinically based recommendations, in comparison to a systematic review. However, the strength of this review is the map of evidence for where men are currently involved in abortion trajectories. It provides a roadmap for future research, and exploration of other areas within the abortion-related care trajectories framework where men are both directly and indirectly involved. Evidence on men emerges from women’s own narratives, with fewer studies including men in their sample, and fewest having men as their primary sample.

The influence that men and boys can exert can directly and indirectly undermine the autonomy of women, girls, and pregnant people, representing a major barrier to universal sexual and reproductive health and rights. The ability of women, girls, and pregnant people to navigate the contextual realities of abortion-related care is too often defined by men, which limits the fundamental right to autonomy and safe, legal, and free choice for people seeking abortions. Future research could consider interrogating the mechanisms, causes, and intentions that drive men’s attitudes and behaviours, to better understand the conditions under which women and pregnant people seek abortions.

## Footnotes

*
https://datahelpdesk.worldbank.org/knowledgebase/articles/906519-world-bank-country-and-lending-groups

## Appendix A: Outline of search terms for EMBASE, PsychINFO, MEDLINE (Ovid), CAB Direct, CINAHL (n=37).

**Table:**

Lead Author / Year	Country / Region	Aims / objectives	Study sample	Study site	Method
Alex-Hart^63^ 2015	Nigeria Sub-Saharan Africa	To evaluate the sexual behaviours of secondary school students in Port Harcourt	1,047 students (537 women, 510 men)	Six public secondary schools	Quantitative
*Summary of results*	Abortion-specific experiences Of the 11% of men who reported a partner ever being pregnant, 26.8% of male respondents assisted their girlfriends in obtaining an abortion. Individual context 48.2% of men were reported to have denied paternity.				
Appiah-Agyekum^70^ 2015	Ghana Sub-Saharan Africa	To explore the factors that influence abortion decisions	142 students (53 men, 89 women)	University of Ghana students	Qualitative
*Summary of results*	Individual context Key determinants of decision making among students were education, religious beliefs, health reasons, financial/economic factors, and family. Less influential were partner's views, societal pressure/stigma, work /career, and peer influence.				
Aziato^65^ 2016	Ghana Sub-Saharan Africa	Gain an understanding of reactions to unplanned adolescent pregnancies in Ghana	15 focus groups with 92 adolescents aged 10-19 who had a recent termination	Public health facilities in Accra, Kumasi, Tamale	Qualitative
*Summary of results*	Abortion-specific experiences In response to pregnancy, girls reported that the character in the vignette would feel sad, alarmed, uncomfortable, not happy and that she might want to terminate the pregnancy. They mentioned that pregnancy and school were not seen as compatible. Respondents suggested that parents might facilitate an abortion. This was focused on disclosure to mothers, but also discussions included fear/concern over the reaction of both parents (e.g. calling the boy to deal with it). Some suggest parents would provide contraceptives to avoid it happening again. Respondents who had partners who obtained medication worried about the safety. Individual context With regards to partner reaction, adolescents suggested it would be shock, surprise, confusion, denial of pregnancy. If the partner was a student or unemployed, they might suggest termination. Male respondents reported that they could deny the pregnancy.				
Bain^71^ 2019	Ghana Sub-Saharan Africa	To understand the adolescent decision-making process and outcome towards pregnancy and abortion	Adolescents aged 13-19 who had at least one pregnancy (n=15), one abortion (n=15) and 23 stakeholders	Jamestown, Accra, Ghana	Qualitative
	Individual context Partners, friends, and family members were the main groups involved in adolescent abortion decision-making. Fathers influenced in a "top-down" manner, having greater decision-making power including threatening to disown the adolescent unless the pregnancy was terminated.				
Challa^43^ 2018	Ghana Sub-Saharan Africa	To explore the social ecological context of adolescent SRH in Ghana	63 women aged 15-24	School and clinic-based sites in Accra and Kumasi	Qualitative
*Summary of results*	Abortion-specific experiences Many women reported keeping pregnancy or abortion a secret from parents to avoid being disowned, abused verbally or physically), or ejected from the home by family.				
Chatchawet^58^ 2010	Thailand East Asia and the Pacific	To gain a greater understanding of the type and amount of support men can offer women obtaining abortions	23 people (12 women and 11 men) who had experienced complications of unwanted pregnancy termination	Three hospital in-patient departments	Qualitative
*Summary of results*	Abortion-specific experiences Men demonstrated accepting some responsibility for the pregnancy termination. Support was demonstrated by searching for information about pregnancy termination; accompanying women to appointments; staying with them during termination. Most men said desire to assist was about ensuring their partners had an efficient and safe termination. Support could also take the form of providing financial assistance needed. Men in the sample reported that they showed support by not leaving their partner during the abortion. This included: being physically close to their partners; waiting nearby, e.g. in front of the room, during the termination of the pregnancies; and, telephoning their partners. Male partners providing support was seen as lessening any emotionally negative experience of abortion by women.				
Che^61^ 2017	China East Asia and the Pacific	To explore perceptions and decision making around contraceptive use, experiences of abortion services, and post-abortion contraceptive decision-making	40 in-depth interviews with women who had experienced abortions and select partners Seven focus groups with men and women	Facilities in urban and rural settings	Qualitative
*Summary of results*	Abortion-specific experiences Men reported being able to accompany their partners and were invited to join in post-abortion family planning discussions. Men considered being involved in these discussions important.				
Coast^72^ 2016	Zambia Sub-Saharan Africa	Analysing care-seeking pathways of women who had either a safe abortion or sought care following an unsafe abortion	112 women who sought care for abortions or post-abortion care	A hospital in Lusaka, Zambia	Qualitative
*Summary of results*	Abortion-specific experiences In accounts of decision-making, women reflected on weighing up the risks, such as the risk of physical harm versus desperation to remove the pregnancy. Financial costs played a role in the timing and complexity of trajectories of abortion; women without independent means faced dilemmas. Individual context Different sources of advice were sought based on different age groups - e.g. adolescents went to peer groups from fear of parental disapproval. Among married women who feared their partner’s reaction, it was harder to seek informed advice.				
Dahlbäck^69^ 2010	Zambia Sub-Saharan Africa	To explore young women's experiences of pregnancy loss	87 young women who had induced abortions (n=34) and spontaneous abortions (n=53)	A hospital in Lusaka, Zambia	Mixed methods
*Summary of results*	Abortion-specific experiences and Individual context Partner factors played a "decisive role" in the final decision-making process to have an abortion. Five partners abandoned their girlfriends and 11 denied paternity. They refused financial and emotional responsibility.				
Freeman^42^ 2017	Zambia Sub-Saharan Africa	To examine men's involvement in women's abortion seeking	71 women who obtained abortions and 41 who obtained post-abortion care	A hospital in Lusaka, Zambia	Qualitative
*Summary of results*	Abortion-specific experiences Some women deliberately excluded men due to fear of men's interference with abortion decisions or fear of their reaction to the pregnancy. Men's active involvement - most influential when acting as shared decision makers, sounding boards, facilitators to obtaining care by paying, arranging, or accompanying a woman. Husbands and boyfriends were most frequently featured in respondents' narratives of men's participation in abortion decision making. Respondents who decided with their partner to abort the pregnancy typically reported that their partner continued to be involved when they obtained services. These men provided emotional support, facilitated abortion by seeking and providing information about where services could be obtained, and accompanied respondents to access care. Most frequently, men supplied the money for transportation and treatment. Individual context Men rejected paternity or the relationship - this was a common reason that women gave for men being absent. Where men were absent, women were more likely to be attending for post-abortion care, while where men knew of their partner’s abortion, the majority of abortions were safe. Younger women were more likely to report partner violence or rejection than older women, although age did not appear to have an impact on involvement.				
Hirz^41^ 2017	Philippines East Asia and the Pacific	To understand men's belief and perception of their roles surrounding unintended pregnancy and induced abortion	15 men for interviews and 43 for focus group discussions	An urban area in the Philippines	Qualitative
*Summary of results*	Abortion-specific experiences Men stated they would feel morally and financially responsible in the event a pregnancy occurred. Men were more nuanced in the responses in in-depth interviews. They recognised that women are fearful of disclosure, that there are physical and social consequences facing women and that a man's decisions would heavily influence abortion outcomes. (Inter)national and sub-national contexts Occurrence of unintended pregnancies was attributed to God's will. Participants in FDGs endorsed belief that induced abortions were a sin. Men expressed frustration at a perceived lack of control over situations regarding pregnancy and induced abortion, and fear that they did not want to commit or be complicit in a sin.				
Izugbara^49^ 2014	Kenya Sub-Saharan Africa	Explore the drivers of women's choices when pregnant	80 women aged 16-49	Nairobi, Kenya	Qualitative
*Summary of results*	Abortion-specific experiences Fear of partner responses led to women keeping their pregnancies a secret. One respondent reported her partner being violent when she disclosed her pregnancy. Men exerted "considerable" influence over the pregnancy trajectory, both to seek an abortion or continue a pregnancy. Some men paid the women to terminate the pregnancy. Individual context Women with unacceptable pregnancies reported abandonment and rejection by male partners and parents. The type of man was important for women as to whether the pregnancy was acceptable (e.g., age, wealth).				
Kalyanwala^57^ 2010	India South Asia	To examine the abortion-related experiences of unmarried women aged 15-24 who obtained abortions	549 women aged 15-24	16 clinics in Janani	Quantitative
*Summary of results*	Abortion-specific experiences 92% of respondents whose partners knew of the pregnancy reported receiving support: 86% reported emotional support, 51% financial support. Other pregnant individuals reported their father's providing financial support. Women who did not receive support from their partner had higher odds of second trimester abortion than those with full partner support. Those who had first trimester abortions compared to second were more likely to receive partner support (95% vs 82%) and have a partner accompany them (78% vs 48%).				
Kalyanwala^56^ 2012	India South Asia	To interrogate the experiences of unmarried young abortion-seekers	549 women aged 15-24, 26 for interview	16 clinics in Janani	Mixed methods
*Summary of results*	Abortion-specific experiences Lack of partner support was reported by only a few women and most had disclosed their pregnancy / abortion. Partners are more likely than any other to provide support. This support can be: deciding on abortion together, emotional support, accompanying to facility, arranging covering costs More women reported not disclosing to their family out of fear of reaction.				
Kumi-Kyereme^50^ 2014	Ghana Sub-Saharan Africa	To examine the key influences in abortion decision-making in Ghana	401 women with records in abortion logbooks	Three abortion service providers	Mixed methods
*Summary of results*	Abortion-specific experiences Overall, 32.67% (n = 131) of the respondents did not seek approval from anyone before receiving an abortion; 54.36% (n = 218) required their partner’s approval; 8.23% (n = 33) consulted with their mother for the decision; and the remaining 4.74% (n = 19) made the abortion decision with role-players categorized as “Others”, which includes friends, siblings, aunts/uncles, employers and mothers-in-law. Men operationalised their role as ‘breadwinners’ during decision-making around pregnancies and abortions. Individual context Knowledge of the law, occupational status, number of children living and level of formal education increased odds of seeking consent of male partners over "others".				
Leone^47^ 2016	Zambia Sub-Saharan Africa	To compare the costs of post-abortion care following unsafe abortion with the costs of safe abortion care	112 women who sought care for abortions or post-abortion care	A hospital in Lusaka, Zambia	Qualitative
*Summary of results*	Abortion-specific experiences 705 of women reported receiving some help, including from husbands or partners, with 50.4% of that help being financial (e.g., money for transport).				
Macleod^75^ 2013	South Africa Sub-Saharan Africa	To study men's constructions of abortions in South Africa	37 articles on abortion and 20 men	University and East London, South Africa	Mixed methods
*Summary of results*	(Inter)national and sub-national contexts Men reported shock at the notion that a woman would terminate a pregnancy without their consent. The 'New Man' discourse of being supportive and attentive was used in discourses by some focus-group discussants to explain how to persuade a woman out of an abortion.				
Marlow^64^ 2019	Ghana Sub-Saharan Africa	To understand what men, know about abortion, why they support their partners, and develop an intervention to improve safe abortion access	11 focus groups of men aged 15-54 (8-12 men in each focus group)	Upper East and Upper West provinces, Ghana	Qualitative
*Summary of results*	Abortion-specific experiences Men reported learning about abortion services from the hospital, friends, and the radio. Some reported arriving to the hospital having previously tried methods. Men reported seeking the services of herbalists and drugs from pharmacists to keep abortions secret from the community. Out of the 11 focus groups, 7 reported supporting women to abort to finishing schooling, 6 if the women had a young child, 5 for mothers’ life, 4 for incest, 3 to care for current family, 2 if pregnancy unplanned and 1 to avoid shame. Whilst men understood that abortions were more safely provided in hospitals, they reported seeking other providers. (Inter)national and sub-national contexts In seven focus groups, men utilised the language of “sin” and that an abortion was “killing” to draw on community norms against abortions.				
Moore^51^ 2011	Uganda Sub-Saharan Africa	To examine men's and women's perspectives on men's involvement in abortion decision-making and seeking post-abortion care	61 women aged 18-60 and 21 men aged 20-50	Kampala and Mbarara, Uganda	Qualitative
*Summary of results*	Abortion-specific experience There were conditions under which some men expressed support, e.g., being involved in the decision making, helping women make doctors' appointments, providing financial support / facilitating transport. Due to secrecy, men talked about not knowing if their partners had abortion complications. Men stated that if a man finds out that the woman terminated a pregnancy without his knowledge, he cannot support her no matter what health problems she experienced. (Inter)national and sub-national contexts Men's responses largely reflect the prevailing socio-cultural norms and values. When questioned generally, male respondents’ status that men are not supportive of women having abortions. Reasons including not agreeing with the practice, belief that the child is a member of society, that the women could die, fear of being arrested, the woman is hiding an affair. Less frequent were costs of abortion and PAC.				
Mwilike^68^ 2018	Tanzania Sub-Saharan Africa	To determine the feasibility of an education programme	15 pregnant adolescents aged 15-19	A health facility in rural Tanzania	Mixed methods
*Summary of results*	Individual context Rejecting and denying paternity had a significant role on women's decisions about whether to abort, particularly for unmarried, pregnant adolescents.				
Nonnenmacher^46^ 2014	Brazil Latin America and the Caribbean	To explore the perception of women in relation to the reactions and behaviour of their partner in abortions	285 women who had miscarriages and 31 women who had abortions	Hospitals in two Brazilian cities	Quantitative
*Summary of results*	Abortion specific experiences Women reported that their male partners were more supportive of spontaneous than induced abortions and they would try to hide the latter from these partners.				
Obiyan^76^ 2014	Nigeria Sub-Saharan Africa	To explore parental involvement in adolescents' sexual and reproductive health education	460 female adolescents for questionnaires, 31 female adolescents and 33 parents for focus group discussions	Yoruba communities in Osun State	Mixed methods
*Summary of results*	(Inter)national and sub-national contexts Male participants believed that single women were more likely to consider abortion than non-single. Men had mixed feelings about whether unintended pregnancies were their responsibility or not. Fathers argued that mothers were closer to their daughters when it came to discussing abortion intentions and that there were gaps in communication between fathers and adolescents.				
Olsson^73^ 2010	Sri Lanka South Asia	To understand experiences of unmarried pregnancy termination seekers to influence future programme development	19 women who had abortions	A health centre, Colombo	Qualitative
*Summary of results*	Individual context Women had various factors that they considered in the decision to seek pregnancy termination: family pressure; partner's qualities and attitude towards pregnancy; economic aspects; own feelings, values and future fertility. Pregnancies and termination occurred in relatively long-lasting relationships - preceding planned marriage - as out of wedlock pregnancy was reported as unacceptable.				
Omidey^52^ 2011	Nigeria Sub-Saharan Africa	To explore whether abortion options were chosen and how they were perceived	17 (10 women, 7 men) interviews, 4 focus group discussions (2 with men, 2 with women)	Local universities and surrounding areas	Qualitative
*Summary of results*	Abortion-specific experiences Women reported being given money by partners and told to seek an abortion, if unmarried. Partners' reactions were significant, as were parent's reactions for women who were single. Fear of repercussions led some women to decide to abort. Male partners played a significant role in determining pregnancy outcomes, including providing financial, material, and emotional support. Individual context Women reported that their concerns over their partner denying their pregnancy led them to seek abortions, including to avoid a known pregnancy not being associated to a “responsible” man.				
Palomino^53^ 2011	Peru Latin America and the Caribbean	To explore participants' individual experiences with reproduction and reproductive decision-making	Interviews with 12 women aged 21-35, 7 men aged 18-37, 2 focus groups with men and 2 with women (33 participants overall)	Metropolitan Lima	Qualitative
*Summary of results*	Abortion-specific experiences Partners were not always the main sources of finances and resources, and an adolescent boy reported that his financial dependence reduced his role in pregnancy decision-making, which was also reported by adolescent women in the study. Individual context Pregnancy-related decisions were not made by the woman alone. Their partner was generally involved, as well as family members. Men and women differed on who had control, with evidence suggesting it ranged between equal decision-making to male controlled decisions. Some respondents reported being coerced to have an abortion by partners or family members, while multiple men argued that they made the decisions on pregnancy outcomes, including abortions. Other men had more equitable views, including that the pregnant woman should decide. For women, age had an impact on their decision-making, with respondents linking being younger or less informed with allowing partners to take control.				
Petitet^62^ 2015	Cambodia East Asia and the Pacific	To examine the implementation and the effects of the distribution of Medabon on women's reproductive choices and practices	10 women, 6 men, 8 health care providers, 4 pill sellers	One site in Takmao and 7 in Phnom Penh	Qualitative
*Summary of results*	Abortion-specific experiences Men knew about different abortion services and learnt about them through newspapers and radios. Four men had accompanied their partners for medical abortion and expressed a desire to help their partners were possible. Three accompanied their partners for PAC.				
Rehnström Loi^67^ 2018	Kenya Sub-Saharan Africa	To explore decision-making preceding induced abortion	9 women aged 19-32	Jaramogi Oginga Odinga Teaching and Referral Hospital (JOOTRH) or Kisumu East District Hospital (KDH) in Kisumu, Kenya	Qualitative
*Summary of results*	Abortion-specific experiences Disclosure of pregnancies to partners was often done to seek financial support. Almost all women expressed that they had a disagreement with their partner and that their fear of possible consequences (including anger, violence, or divorce) was a factor in the decision to seek care without telling a partner. Women reporting feeling forced or misled into abortions, with one respondent reporting that her partner involved an abortion provider to help him terminate the pregnancy without her consent. Individual context Women reported that their partners’ unwillingness to financially support a child was a key reason for seeking abortions. In addition, unstable relationships with partners were cited. The context of the relationship of the woman and the man responsible for the pregnancy also influenced disclosure - women who were single were more likely not to tell their partner of the pregnancy or abortion.				
Rominski^44^ 2017	Ghana Sub-Saharan Africa	To understand the perspective of women who decide to terminate	18 women seeking care for complications from abortions and 11 for abortion-care (aged 13-35)	Three hospitals in Ghana	Qualitative
*Summary of results*	Abortion-specific experiences Women reported that they self-managed their abortion over fear of disclosure. Women learnt abortion methods through social networks. They expressed taking drugs provided by friends or boyfriends, despite not necessarily knowing what they were.				
Schwandt^45^ 2013	Ghana Sub-Saharan Africa	To understand the decision-making process associated with induced abortion in Ghana	58 interviews (19 with men, 20 with women, 11 with family planning nurses, 8 obstetricians / gynaecologists) and 9 focus groups (4 with women, 2 with men, 1 with family planning nurses, 2 with obstetricians / gynaecologists)	two teaching hospitals, Ghana	Qualitative
*Summary of results*	Abortion-specific experiences Women discussed fears disclosing and some did not disclose prior to abortion over fear of reaction. Individual context Men were the first decision makers post pregnancy discovery. Their acceptance or rejection was critical - acceptance was of paternity. Men's ability to deny responsibility was a major fear of respondents. This has an indirect impact on the abortion trajectory of a woman				
Shekhar^40^ 2010	Kyrgyzstan Europe and Central Asia	To estimate the abortion rates by different background characteristics	3848 women aged 15-49 (Demographic and Health Survey)	National	Quantitative
*Summary of results*	Individual context Women's attitude towards becoming pregnant and their husband's attitude towards abortion were significantly associated with the likelihood of an induced abortion.				
Sowmini^55^ 2013	India South Asia	To identify the reasons that cause delay for adolescents and young women seeking safe abortion services	34 unmarried young women seeking abortion	Tertiary hospital abortion clinic, Trivandrum	Qualitative
	Abortion-specific experiences Most adolescents were accompanied by their mothers to obtain an abortion, with few involving their sexual partners and only two reported that their partner accompanied them or provided financial support.				
Srivastava^66^ 2019	India South Asia		20 medical abortion users and 20 partners	Three districts, Uttar Pradesh, India	Qualitative
*Summary of results*	Abortion-specific experiences Men were frequently the ones bringing MA kits for their female partners. Male respondents indicated that the chemist was often a male friend of theirs. Lack of knowledge meant the chemist was often trusted to provide the right information and dosage, as well as potential side effects. Such information could be lost in transit when male partners obtained the abortion method on behalf of their partner, leading to a lack of knowledge of side effects amongst women.				
Steven^74^ 2019	Democratic Republic of the Congo Sub-Saharan Africa	To explore leaders' perceptions of their role in addressing unintended pregnancies in the community	12 male community leaders	Six rural health zones, North and South Kivu	Qualitative
*Summary of results*	(Inter)national and sub-national contexts Community leaders were all male. Their attitudes towards abortion were very negative, including perceiving abortion as unchristian, immoral, or in violation of community norms. Women who had abortions were seen as criminals, and community leaders reported involving the police in instances of abortions or isolating / forcing a woman out of the community. In spite of this, community leaders indicated that women who had an abusive, alcoholic, or unemployed partner, or who faced financial difficulties, could seek an abortion. Community leaders considered themselves responsible for the provision of PAC.				
Tatum^48^ 2012	Mexico Latin America and the Caribbean	To examine the factors influencing how young women make reproductive decisions	12 interviews and 4 focus groups with women aged 13 to 17	Mexico City	Qualitative
*Summary of results*	Abortion-specific experiences Partners could offer emotional support, though often support was in accordance with the partner's wishes and not necessarily the respondent’s. Of the six interview respondents who had an abortion, four reported that their partner was willing to assume responsibility of fatherhood, including involving the adolescent’s father for approval. Two respondents described being forced to have an abortion by their fathers without their consent. Individual context Absence of a viable co-parent influenced some women to abort. In all cases where partner was not involved, women decided to abort. For the two focus group discussion participants who did not have an abortion, their partners assumed responsibility and were working.				
Tong^59^ 2014	Malaysia East Asia and the Pacific	To explore the experiences of women and their needs regarding abortion	31 women aged 21-43 who had obtained abortions	An urban family planning clinic in Penang	Qualitative
*Summary of results*	Abortion-specific experiences Some respondents indicated that an abortion decision should be between a woman and her partner. Others reported that they felt forced to abort as their partner claimed not to be ready for marriage or to financially support the child, thus making the pregnancy unacceptable. Partners could play a supportive role, including seeking information, paying for services and accompanying women.				
Ushie^54^ 2019	Kenya Sub-Saharan Africa	To understand community perception of abortion is critical in informing the design and delivery of interventions to increase access to safe abortion	36 women and 12 men for interview, 9 health care workers. 9 focus groups with women and 9 with men	Kisumu and Nairobi counties	Qualitative
*Summary of results*	Abortion-specific experiences The study reported that the majority of girls relied on boyfriends, as well as friends, relatives, and mothers, to raise money for their care. This includes one (male) respondent referencing that this could result in unwanted disclosure of a pregnancy. (Inter)national and sub-national contexts In communities where men, and their families, conduct informal “background checks” on women, knowledge of abortions is considered undesirable and means a woman is perceived as a bad potential wife. Men report thinking that these women might continue to have abortions, which limits their ability to achieve social success through parenting. However, men and women also reported that abortions were increasingly normalised in their communities.				
Zuo^60^ 2015	China East Asia and the Pacific	To examine why unmarried women delay obtaining an abortion and identify correlates of the delayed decision	1,271 unmarried women aged 15-24 who had sought abortions	Shangai, Chengdu, and Taiyun	Quantitative
*Summary of results*	Abortion-specific experiences 73-85% of male partners had positive reactions to pregnancy disclosures and provided comfort / solutions. 12-28% of women reported that partners were pleased about the pregnancy, either due to it cementing their relationship or to confirming fertility. 6-12% of partners responded with fear.				
